# Designing New Antibacterial Wound Dressings: Development of a Dual Layer Cotton Material Coated with Poly(Vinyl Alcohol)_Chitosan Nanofibers Incorporating *Agrimonia eupatoria* L. Extract

**DOI:** 10.3390/molecules26010083

**Published:** 2020-12-27

**Authors:** Cláudia Mouro, Colum P. Dunne, Isabel C. Gouveia

**Affiliations:** 1 FibEnTech Research Unit, Textile Department, Faculty of Engineering, University of Beira Interior, 6201-001 Covilhã, Portugal; d1684@ubi.pt; 2 Centre for Interventions in Infection, Inflammation & Immunity (4i), School of Medicine, University of Limerick, V94 T9PX Limerick, Ireland; colum.dunne@ul.ie

**Keywords:** cotton, nanofibers, electrospinning, *Agrimonia eupatoria* L., antibacterial wound dressings, skin infections

## Abstract

Wounds display particular vulnerability to microbial invasion and infections by pathogenic bacteria. Therefore, to reduce the risk of wound infections, researchers have expended considerable energy on developing advanced therapeutic dressings, such as electrospun membranes containing antimicrobial agents. Among the most used antimicrobial agents, medicinal plant extracts demonstrate considerable potential for clinical use, due primarily to their efficacy allied to relatively low incidence of adverse side-effects. In this context, the present work aimed to develop a unique dual-layer composite material with enhanced antibacterial activity derived from a coating layer of Poly(vinyl alcohol) (PVA) and Chitosan (CS) containing *Agrimonia eupatoria* L. (AG). This novel material has properties that facilitate it being electrospun above a conventional cotton gauze bandage pre-treated with 2,2,6,6-tetramethylpiperidinyl-1-oxy free radical (TEMPO). The produced dual-layer composite material demonstrated features attractive in production of wound dressings, specifically, wettability, porosity, and swelling capacity. Moreover, antibacterial assays showed that AG-incorporated into PVA_CS’s coating layer could effectively inhibit *Staphylococcus aureus* (*S. aureus*) and *Pseudomonas aeruginosa* (*P. aeruginosa*) growth. Equally important, the cytotoxic profile of the dual-layer material in normal human dermal fibroblast (NHDF) cells demonstrated biocompatibility. In summary, these data provide initial confidence that the TEMPO-oxidized cotton/PVA_CS dressing material containing AG extract demonstrates adequate mechanical attributes for use as a wound dressing and represents a promising approach to prevention of bacterial wound contamination.

## 1. Introduction

Although skin is a relatively robust protection against infectious agents [1,2], it is prone to compromise by physical damage or disease-related lesions that affect both the structure and function. Of these, cutaneous wounds represent a favorable microenvironment for bacterial colonization and proliferation, facilitating occurrence of infections especially in patients with impaired immune responses [3,4,5,6,7]. In an era of increasing multidrug-resistance, impaired treatment options, and elevated rates of morbidity and mortality [6], the development of wound dressings with intrinsic antimicrobial properties is increasingly desirable.

Traditionally, wound dressings comprise gauzes, films, foams, hydrogels, and hydrocolloids and these have been used to cover the wound site to improve the healing process [3,6,7,8]. Nevertheless, natural textile materials (such as cotton) continue to be most used due to their low cost, limited adverse effects, easy handling and fabrication, and adequate mechanical support [8]. However, such textile wound dressings can lead to wound dehydration, adherence to wound beds in the presence of high-exudate wounds, and cause trauma and pain during removal [8]. Due to this, composite wound dressings, which combine the most advantageous features of these different materials have been developed to prevent bacterial infections while enhancing the healing process. Likewise, complementary approaches have involved innovative dressings with intrinsic antibacterial activity, integrated extrinsic antimicrobial agents, surface modification, and topical application have been explored [3,9,10,11,12]. However, the latter approach involving topical administration has proved problematic due to subsequent irritation or allergic contact dermatitis reactions that negatively impact the healing process [11,13]. Furthermore, non-healing, chronic wounds often present with considerable volumes of exudate, which can reduce the penetration rate of topically administered agents [14]. In efforts to overcome these challenges, attention has turned to the potential use of nanofiber materials produced from naturally derived or synthetic biopolymer mixtures that undergo electrospinning, with enhanced capability to promote skin regeneration while conferring protection against bacterial infection [10,15,16]. These beneficial traits are mediated somewhat through a physical similarity of the electrospun nanofibrous membranes to the native skin extracellular matrix that promotes cell adhesion and proliferation allied with the ability to deliver therapeutic and bioactive compounds directly to the wound site [10,15,16]. In addition, the simplicity, cost-effectiveness, and versatility of the electrospinning, as well as the unique features of the produced electrospun nanofibers, make this technique attractive for wound dressing applications [10,15,16].

Among the therapeutic and bioactive compounds, many are sourced from medicinal plants [10,16]. More specifically, crude plant extracts that (presumably) contain mixtures of bioactive compounds have demonstrated attractive levels of efficacy that is often greater than those exhibited by single isolated compounds from those same plants. This efficacy is likely to relate to synergic interactions between constituent compounds or possible protection of bioactive agents from enzymatic degradation during extraction processes [16,17,18,19]. One such crude extract derives from *Agrimonia eupatoria* L., a member of the family Rosaceae, commonly known as agrimony (AG) has been analyzed and shown to be rich in tannins, flavonoids, phenolic acids, and triterpenoids with recognized anti-inflammatory, antioxidant, antimicrobial, and antibiofilm properties [20,21,22,23].

In this study, a new composite wound dressing material was produced that comprised two distinct layers. To accomplish that, a cotton gauze bandage that had been oxidized previously with the 2,2,6,6-tetramethylpiperidine-1-oxyl radical (TEMPO)/sodium bromide (NaBr)/sodium hypochlorite (NaClO) system was used as a substrate for the nanofibrous membrane composed of Poly(vinyl alcohol) (PVA), Chitosan (CS), and AG. Hence, the produced electrospun PVA_CS membrane combined with AG is intended to reduce the adhesion behavior of the textile dressing when applied to the wound bed and to improve the healing process, complemented by suppression of wound infection mediated by inherent antibacterial activity.

## 2. Results and Discussion

### 2.1. Minimum Inhibitory Concentration (MIC)

**Figure 1:**
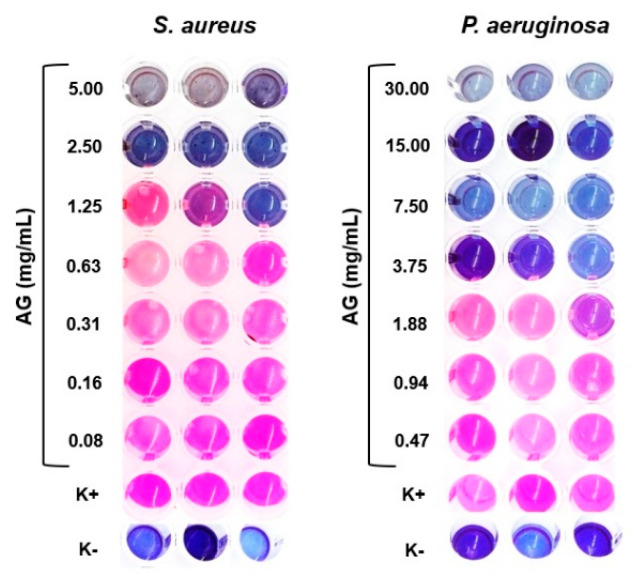
Determination of the minimum inhibitory concentration (MIC) against *Staphylococcus aureus* (*S. aureus*) and *Pseudomonas aeruginosa* (*P. aeruginosa*) by resazurin-based 96-well plate microdilution method.

The in vitro antibacterial efficiency measures of the ethanol crude extract of AG against *Staphylococcus aureus* (*S. aureus*) and *Pseudomonas aeruginosa* (*P. aeruginosa*) were found to be 1.25 and 3.75 mg/mL, respectively, Figure 1. These results suggest that the crude AG extract is less effective against Gram-negative bacteria (*P. aeruginosa*) compared to Gram-positive (*S. aureus*), albeit that only two species were studied. Notably, a similar effect was described previously by Muruzovic et al., where MIC values of 1.25 mg/mL and 0.62 mg/mL were obtained for *P. aeruginosa* and *S. aureus*, respectively [23]. However, there is a caveat in that the geographic location, the season in which the medicinal plants are harvested, and the chosen extraction method may impact the eventual extract’s chemical composition and, thereby, MIC values.

### 2.2. Characterization of the Dual-Layer Dressing Materials

#### 2.2.1. Assessment of the Morphologic Features

**Figure 2:**
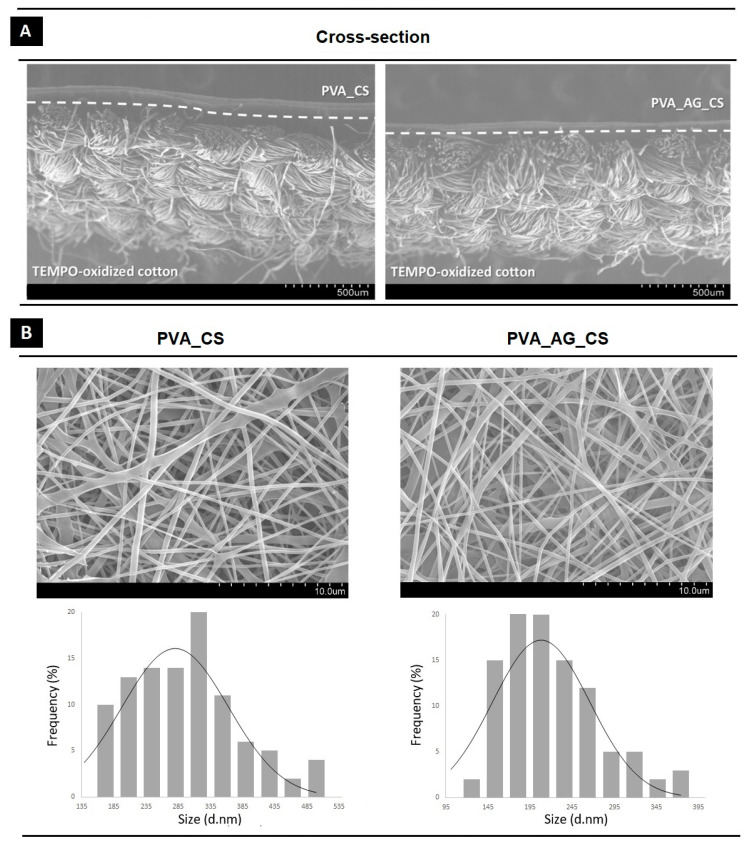
Morphological characterization of the produced 2,2,6,6-tetramethylpiperidine-1-oxyl radical (TEMPO)-oxidized cotton/ Poly(vinyl alcohol) (PVA)_Chitosan (CS) materials with and without crude *Agrimonia eupatoria* L. (AG) extract. SEM images of the cross-sections (**A**); surface morphologies and diameter distribution of the coating layers of PVA_CS and PVA_AG_CS nanofibers (**B**).

The TEMPO-oxidized cotton/PVA_CS materials with and without incorporated crude AG extract were successfully fabricated with a bilayer organization as demonstrated by the cross-sectional SEM images, Figure 2A. Additionally, the coating layers of PVA_CS and PVA_AG_CS were electrospun directly over the cotton bandage gauze to enable absorption of wound exudates in a controlled manner, to maintain a suitable moist environment, and to ensure proper nutrient and gas exchange. This electrospun membrane proved to be non-toxic, biocompatible and biodegradable, with antimicrobial ability to enhance collagen synthesis, as previously reported membranes produced with PVA and CS [24]. Moreover, incorporation of the crude AG extract into this layer supplements the antimicrobial activity on the dual-layer material. With regard to physical properties specifically, the diameter of the coating layer’s nanofibers was determined by SEM imaging, Figure 2B. Uniform and homogeneous nanofibers that have an average diameter of 280.20 ± 82.65 nm and 208.11 ± 57.92 nm were obtained for CS_PVA and CS_AG_PVA, respectively. These diameters are within the size range displayed by collagen fibers in natural skin (50–400 nm), potentially promoting cell adhesion, migration, and proliferation [25,26].

#### 2.2.2. Fourier Transform Infrared Spectroscopy (ATR-FTIR)

**Figure 3:**
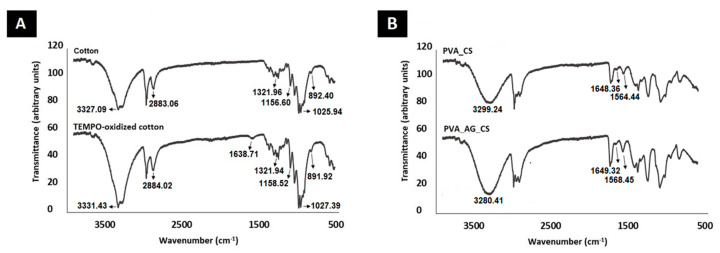
Attenuated total reflection-Fourier transform infrared (ATR-FTIR) analysis of the produced dual-layer dressing materials. Cotton layer (**A**), and the nanofibrous coating layers (**B**).

The ATR-FTIR spectra were acquired to characterize the chemical composition of the produced nanocomposite wound dressing materials, Figure 3. The spectrum of the TEMPO-oxidized cotton reflected the characteristic peaks of unmodified cotton at around 3330 cm^−1^ (O–H stretching vibration), 2880 cm^−1^ (C–H stretching vibration), 1330 cm^−1^ (O–H deformation vibration), 1160 and 890 cm^−1^ (β-(1–4) glycoside bridge), and 1030 cm^−1^ (C–O–C stretching vibration). In addition, in the TEMPO-oxidized cotton spectrum, it is possible to detect a band at about 1600 cm^−1^ (asymmetrical COO^−^ stretching vibration) Figure 3A. This result is associated with the TEMPO-mediated oxidation conditions, which impart a negative surface charge to the cellulose cotton [27]. In comparison, the spectrum of the coated layer displays the characteristic bands of CS around 1560 cm^−1^ (N–H bending vibrations) and 1650 cm^−1^ (C=O stretching vibrations), as well as the typical broad peak of PVA_CS in the region between 3000 and 3500 cm^−1^ (–OH and –NH stretching) Figure 3B. The presence of these bands is evidence of the effective blending of the PVA_CS. Furthermore, an increase in the intensity of the PVA_AG_CS’s characteristic peaks demonstrated the successful blending of the crude AG extract into the PVA_CS nanofibers. A similar effect was described by Hadisi et al., whereby the characteristic peaks of henna overlapped with the peaks of gelatin-oxidized starch nanofibers [28].

### 2.2.3. Mechanical Strength Behavior

In order to confirm whether the produced dual-layer dressing materials are suitable for support of mechanisms involved in the healing process, the mechanical behavior of the TEMPO-oxidized cotton/PVA_CS with and without crude AG extract was determined in dry conditions from tensile strength, Young’s modulus, and elongation at break, Table 1. The mechanical properties of the TEMPO-oxidized cotton, PVA_CS, and PVA_AG_CS nanofibers were also evaluated for further comparison purposes.

The TEMPO-oxidized cotton/PVA_CS displayed a tensile strength value of 22.48 ± 4.46 MPa, whereas the TEMPO-oxidized cotton/PVA_AG_CS exhibited a value of 26.55 ± 1.41 MPa. These results, and in comparison with the tensile strength obtained for the individual layers, confirmed that an appropriate balance is required between the higher mechanical strength of the TEMPO-oxidized cotton and the weaker mechanical behavior exhibited by the PVA_CS and PVA_AG_CS nanofibrous coating layers. That requirement mirrors the tensile strengths’ range of 5.00 to 30.00 MPa associated with native skin [2].

TEMPO-oxidized cotton/PVA_CS with and without crude AG extract demonstrated a similar Young’s modulus of elasticity of 0.46 ± 0.11 GPa and 0.40 ± 0.14 GPa, respectively. The elongation at break assays determined that TEMPO-oxidized cotton/PVA_CS and TEMPO-oxidized cotton/PVA_AG_CS materials bore a similar strain of 5.80 ± 0.89% and 5.95 ± 1.71%, respectively. While these values are outside the range available for native skin (Young’s modulus ranging between 0.4 and 20 MPa and elongations ranging from 10% to 115%) [2], other potential wound dressing materials (previously described) have similarly high Young’s modulus (e.g., ranging between 278.6 ± 12.5 MPa and 456.2 ± 22.5 MPa) [29].

### 2.2.4. Wetting Studies

Water droplet contact angle (WCA) between 40° and 70° has been considered as a favorable surface in contrast to very hydrophilic (WCA < 20°) and hydrophobic (WCA > 90°) surfaces [30,31]. This relates to cell adhesion, migration, and proliferation, and absorption of excess wound exudate.

In this study, the TEMPO-oxidized cotton displayed a WCA value of 27.53 ± 6.84°, confirming its highly hydrophilic nature [27]. This result is indicative of the ability of the hydroxyl groups present on the cotton fabric to absorb fluids. However, it is important to remember that although the cotton bandage gauzes could be used for absorbing exudates, their efficiency can be lost when these dressings become saturated, resulting in tissue maceration and potential contribution to increase in the occurrence of infections [10].

Notably, the coating layer of PVA_CS exhibited a WCA value of 54.03 ± 11.98°, and the PVA_AG_CS layer had a WCA score of 42.37 ± 7.52°. These results demonstrated that the presence of polar phytochemicals in the crude AG extract improves the wettability of the PVA_CS, creating an effective moist environment, which is essential to the healing process [32].

### 2.2.5. Porosity Assessment

Ideally, the dual dressing materials’ porosity should allow proper gas exchange, efficient water and nutrients supply, and effective fluid absorption while maintaining the moisture balance at the wound site [10]. High porosity is also expected to support cell accommodation and migration. In that context, porosity values within the range of 60–90% have been found essential for effective healing processes [10]. In our study, the TEMPO-oxidized cotton exhibited the lowest porosity value (76.09 ± 5.64%), whereas the blend of PVA_CS and PVA_AG_CS scored highest porosity values (84.09 ± 6.71% and 92.77 ± 6.01%, respectively). These findings indicate that nanofibers with lower diameters, like PVA_AG_CS (208.11 ± 57.92 nm), lead to a higher number of void spaces available between nanofibers and result in higher porosity values. This physical feature also provides a larger specific surface area suitable for cell adhesion and growth during the healing process.

### 2.2.6. Water Vapor Transmission Rates (WVTRs)

An ideal wound dressing should keep the wound moist and also absorb excess fluids, while allowing air and vapor permeation. Specifically, water vapor transmission rates (WVTRs) ranging between 2000 and 2500 g/m^2^/day facilitate water vapor exchanges and prevent exudate accumulation, which can contribute to the breakdown of the skin extracellular matrix (ECM) components or provoke skin maceration of the healthy tissue surrounding the wound [10]. In this study, the TEMPO-oxidized cotton coated with a blend of PVA_CS without and with crude AG extract had similar WVTR values of 1246.75 ± 148.63 g/m^2^/day and 1304.09 ± 123.13 g/m^2^/day, respectively.

These results suggest that the obtained WVTRs are outside of the ideal range for a proper wound dressing material. However, it is interesting that our test coatings performed better than several commercially available wound dressings (Comfeel (Coloplast A/s) (308 g/m^2^/day), Dermiflex (Johnson & Johnson, New Brunswick, Nova Jersey, EUA) (90 g/m^2^/day), and Duroderm (ConvaTec Ltd., Deeside, Flintshire, UK) (886 ± 32 g/m^2^/day)) [33].

### 2.2.7. Swelling and In Vitro Degradation Studies

**Figure 4:**
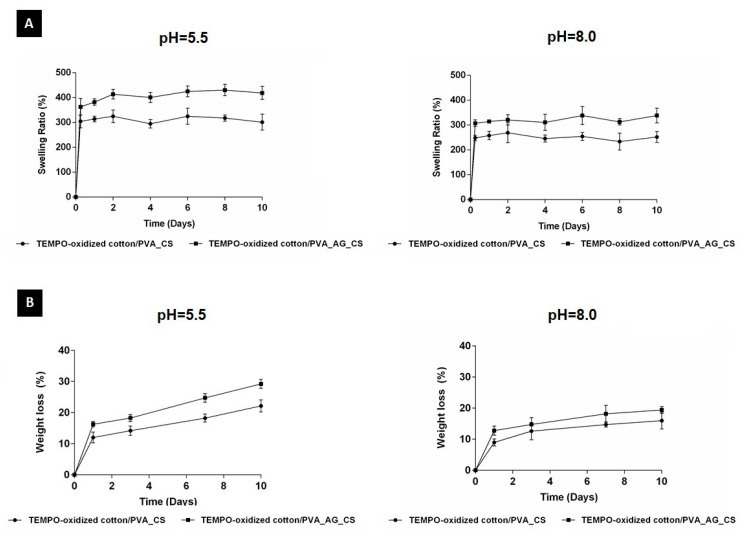
Characterization of the TEMPO-oxidized cotton/PVA_CS materials with and without crude AG extract incorporated. Swelling profile (**A**); weight loss at pH = 5.5 and pH = 8.0 (**B**).

The water uptake and weight loss tests of the our dual-layer dressing materials were performed over 10 days in PBS buffer at pH = 5.5 and pH = 8.0 to mimic the acidic wound environment favorable for wound healing, and the attributes of wound fluid, respectively, Figure 4A,B. The TEMPO-oxidized cotton/PVA_CS displayed a lower degree of swelling at pH = 5.5 and pH = 8.0 of ~310% and ~250%, respectively. The TEMPO-oxidized cotton/PVA_CS containing the crude AG extract presented a water absorption ratio at pH = 5.5 and pH = 8.0 of ~400% and ~320%, respectively, Figure 4A. These behaviors can be explained by the higher hydrophilic character of the electrospun PVA_AG_CS nanofibers. In addition, the highest swelling capacity was observed at pH = 5.5, due to the protonation of the amino NH_2_ and acetamido CH_3_C(O)NH groups of CS, which can form ammonium cations and increase the degree of swelling [34]. Hence, it was evident that TEMPO-oxidized cotton/PVA_AG_CS at both pHs provided a more appropriate environment for exudates absorption.

The TEMPO-oxidized cotton/PVA_CS displayed weight losses of 16.63 ± 4.49% and 13.05 ± 3.07% at pH = 5.5 and pH = 8.0, while TEMPO-oxidized cotton/PVA_AG_CS underwent weight loss of 22.12 ± 5.96% and 16.25 ± 3.07% at pH = 5.5 and pH = 8.0, respectively, Figure 4B. This is mainly due to the addition of the biodegradable and natural components, such as PVA, CS, and crude AG plant extract to the non-degradable TEMPO-oxidized cotton. It is reasonable to conclude that the components present in the coating layer are the most important regulators of the degradation profile, which itself must be similar to the skin regeneration rate [35]. Nevertheless, the produced dual-layer dressing materials showed no significant differences in the hydrolytic degradation.

## 2.3. Study of the In Vitro AG Release

**Figure 5:**
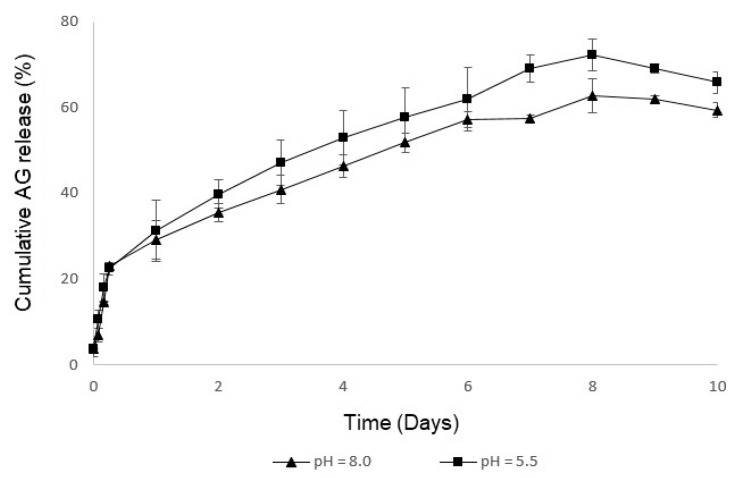
Evaluation of the in vitro release profile of the crude AG extract incorporated into the TEMPO-oxidized cotton/PVA_CS material.

The release behavior of the nanofibers was investigated by immersing the produced material in PBS at pH = 5.5 (pH found on normal skin and beneficial for wound healing) and pH = 8.0 (pH of wound exudate), for 10 days, Figure 5. The result was an initial burst release observed within the first 6 h, followed by a sustained diffusion or slow release. Correspondingly, a cumulative release of the AG extract from the lower layer was 72.18 ± 3.71% and 62.68 ± 3.87% at pH = 5.5 and pH = 8.0, respectively. This behavior can be explained by the higher swelling capacity of the TEMPO-oxidized cotton/PVA_AG_CS dressing material at pH = 5.5, as well as weight loss. Hence, it appeared that the AG release rate from PVA_CS is mainly controlled by the degree of swelling and weight loss rate, being the cumulative release of the crude AG extract crucial if occurrence of infections at the wound site is to be avoided.

## 2.4. Antibacterial Properties of the Dual-Layer Dressing Materials

In this study, the antimicrobial properties were characterized against *S. aureus* (Gram-positive) and *P. aeruginosa* (Gram-negative), the bacteria most commonly isolated from wound infections [7,36].

**Figure 6:**
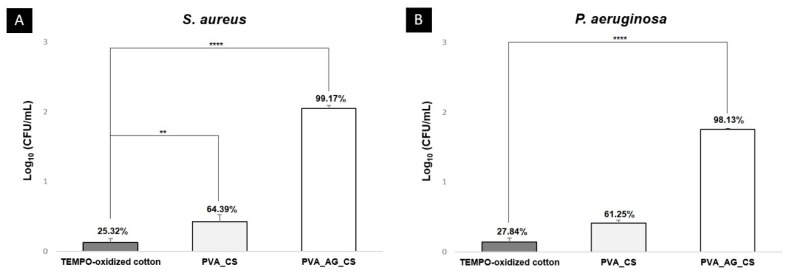
Evaluation of the antibacterial properties of the TEMPO-oxidized cotton, PVA_CS, and PVA_AG_CS nanofibers against *S. aureus* (**A**); *P. aeruginosa* (**B**). Data are represented as average ± standard deviation (S.D.), ** *p* < 0.001 and **** *p* < 0.0001).

The observed results (Figure 6) show an inhibitory effect after 24 h of contact of 25.32 ± 5.62% and 27.84 ± 5.77% for the TEMPO-oxidized cotton sample against *S. aureus* and *P. aeruginosa*, respectively. This suggests that the cellulose cotton may help to prevent bacterial penetration into the wound, while offering mechanical support to the wound site. In contrast, the coating layer of PVA_AG_CS exhibited a higher inhibitory effect, 99.17 ± 4.05% and 98.13 ± 0.88% for *S. aureus* and *P. aeruginosa*, while the coating layer of PVA_CS inhibited *S. aureus* and *P. aeruginosa* growth to lesser degrees (64.39 ± 10.07% and 61.25 ± 4.22%*,* respectively) (Figure 6). These values reflect the intrinsic bactericidal activity of CS and crude AG extract and previous studies demonstrating that inhibitory or anti-adhesion attributes of secondary metabolites, such as flavonoids, phenolic acids, and triterpenoids of AG [21,23]. In addition, the cationic properties of CS allow it to interact with the negatively charged bacterial material surface, resulting in increased cell wall permeability, and consequent disruption and loss of intracellular components [37,38].

## 2.5. In Vitro Cytotoxicity Assay (MTT)

**Figure 7:**
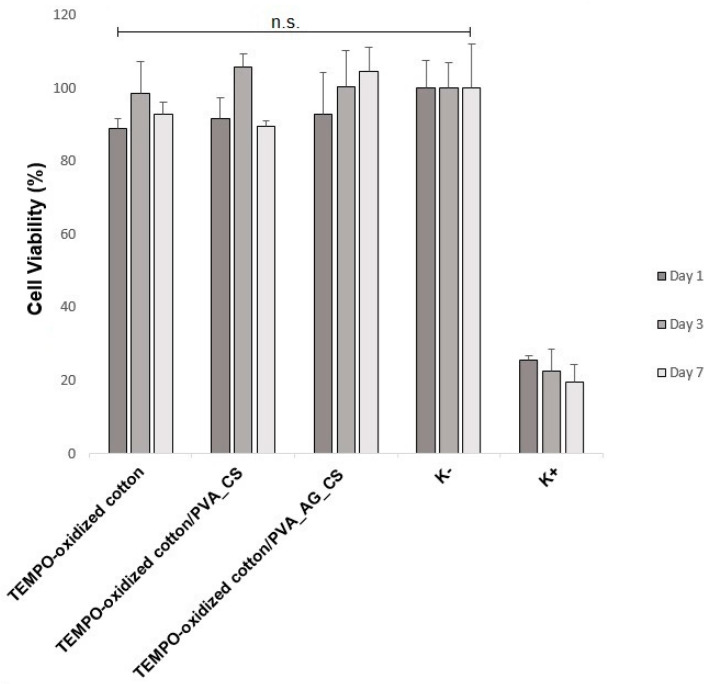
Evaluation of the normal human dermal fibroblast (NHDF) cell viability after 1, 3, and 7 days in contact with the TEMPO-oxidized cotton and the produced dual-layer dressing materials.

An ideal wound dressing should be biocompatible and play a major role in cellular interactions, supporting the healing process. To explore this aspect of our coatings, the normal human dermal fibroblast (NHDF) cells were chosen as implicated in producing new ECM components, and collagen fibers, which are integral events in cell migration and proliferation responsible for reestablishment of damaged tissue [7,10,36]. As shown in Figure 7, the chosen direct MTT (3-(4,5-Dimethyl-2-thiazolyl)-2,5-diphenyl-2H-tetrazolium bromide) cytocompatibility assay provided no evidence of cytotoxicity against NHDF cells. Notably, the produced dual-layer dressing materials displayed more than 80% cell viability, even after 7 days. Additionally, the TEMPO-oxidized cotton coated with PVA_AG_CS exhibited an ascending effect of proliferation from 1 to 7 days. This trend could be attributed to the higher porosity and hydrophilic character exhibited by the PVA_AG_CS nanofibers, which are considered to be proper for encouraging the cell attachment and proliferation at the nanofibrous membrane’s surface.

Moreover, the toxicity levels of the produced dual-layer dressing materials were determined from the standard percentage cell viability ranges (as seen in Table 2). The obtained results showed that the tested materials displayed toxicity levels between 0 and 1, which are considered as nontoxic to the human body [39].

## 3. Materials and Methods

### 3.1. Materials

*Agrimonia eupatoria* L. (AG) plant was obtained (CHÁ HUNOS, Lda., Vila Nova de Gaia, Portugal), stored and used according to the supplier’s recommendations. Normal human dermal fibroblast (NHDF) cells were acquired from ATCC—American Type Culture Collection. Cotton fabric was obtained from James H. Heal & Co. Ltd. (Halifax, UK). Resazurin (7-hydroxy-3*H*-phenoxazin-3-one 10-oxide) dye, 2,2,6,6-tetramethylpiperidine-1-oxyl radical (TEMPO), sodium bromide (NaBr), sodium hypochlorite (NaOCl), chitosan (CS) (low molecular weight), sodium hydroxide (NaOH), hydrochloric acid (HCl), sodium chloride (NaCl), Mueller-Hinton broth (MHB), tween 80, dimethyl sulfoxide (DMSO) anhydrous ≥ 99.9%, trypsin, and 3-(4,5-Dimethyl-2-thiazolyl)-2,5-diphenyl-2H-tetrazolium bromide (MTT) were purchased from Sigma-Aldrich (Sintra, Portugal). Glacial acetic acid and ethanol absolute were acquired from Fisher Scientific (Porto Salvo, Portugal). Poly(vinyl alcohol) (PVA) (MW 115.000 g/mol) was purchased from VWR International (Carnaxide, Portugal). Nutrient agar (NA), nutrient broth (NB), and agar for microbiology were bought from Fluka (Sintra, Portugal). Brain Heart Infusion (BHI) broth was obtained from Panreac (Barcelona, Spain). Phosphate-buffered saline (PBS) was purchased from Alfa Aesar (Ward Hill, USA). All solvents were of analytical grade and used as received without further purification.

### 3.2. Methods

#### 3.2.1. Preparation of Crude AG Extract

The dried aerial parts of the medicinal AG plant (CHÁ HUNOS, Lda., Vila Nova de Gaia, Portugal) were powdered and subjected to cold maceration with ethanol/water in a ratio of 80:20 for 24 h. The extracted solution was filtered through a membrane filter (Whatman No. 1, 11 µm pore size), and the filtrate solvent evaporated using a Rotavapor apparatus (Buchi Rotavapor RE 111). The obtained crude AG extract was used throughout the study.

#### 3.2.2. Minimum Inhibitory Concentration (MIC)

Minimum inhibitory concentration (MIC) of the crude AG extract was evaluated against *S. aureus* (ATTC 6538) and *P. aeruginosa* (PA25) using the broth microdilution method according to the CLSI NCLS M7-A6 guidelines. Briefly, stock solutions of the AG were prepared (30 to 0.08 mg/mL) in sterile MHB. Then, 50 µL of each AG dilution was aliquoted in 96 multi-well polystyrene plates (Sigma-Aldrich) followed by 50 µL bacterial inoculum containing ~10^7^ colony-forming unit (CFU)/mL. The plates were incubated at 37 °C for 18–24 h. After incubation, 30 µL of 0.02% resazurin solution was added to each microplate well to aid visualization of bacterial growth. The plates were further incubated for 2–4 h for the observation of color change. The lowest concentration at which the blue-purple resazurin color (no bacteria growth) changed to pink (bacteria growth) was taken as the MIC value. The MIC values was defined as the lowest concentration of the AG extract resulting in complete inhibition of visible growth. AG-free wells only with MHB medium and inoculum were used as a positive control (K^+^), whereas wells containing only MHB medium were used as a negative control (K^−^).

#### 3.2.3. Fabrication of the Dual-Layer Dressing Materials

First layer: Firstly, cotton fabric was pre-activated with TEMPO to provide a negative net electrostatic charge. To accomplish this, a solution of 0.0125% (*w/v*) TEMPO, 0.125% (*w/v*) NaBr, and 3.2% (*v/v*) NaClO was prepared, and its pH value was adjusted to 10.5 with 1 N NaOH. Then, 2 g of cellulose cotton was immersed in 50 mL of the TEMPO solution and stirred for 60 min. The pH of the solution was reduced to 7 using 0.1 M HCl, and the cotton fabric washed in deionized water. After the activation process, the second layer was electrospun directly over the first layer to produce the dual-layer materials.

Second layer: A blend comprising 10% (*w/v*) PVA (well-known fiber-forming polymer) and 2% (*w/v*) CS (natural cationic polysaccharide) was prepared in water and 0.1 M CH_3_COOH, respectively. The pH of the CS solution was adjusted to 5 using 0.1 M HCl, resulting in a positive electrostatic charge, and added to the PVA solution in a ratio of 30:70. This blend with and without 5.0 *wt.*% crude AG extract was electrospun on top of the TEMPO-oxidized cotton using Nanospider^TM^ electrospinning (Nanospider laboratory machine NS LAB 500S from Elmarco S.R.O., Liberec, Czech Republic) at an electrode rotation of 55 Hz, using a working distance of 13 cm, and an applied voltage of 75 kV, during ~1 h.

The raw PVA_CS and PVA_AG_CS were collected on polypropylene nonwoven fabric over the same conditions.

#### 3.2.4. Characterization of the Dual-Layer Dressing Materials

##### Assessment of the Morphologic Features

The morphology of the dual-layer dressing materials was determined by scanning electron microscopy (SEM, S-3400N, Hitachi, Tokyo, Japan) at an accelerating voltage of 20 kV. Before imaging, a small section of the nanofibers coating layer was placed on SEM specimen stubs, and then sputter coating with a thin layer of gold using the Quorum Q150R ES sputter coater (Quorum Technologies Ltd., Laughton, East Sussex, UK) to make them electrically conductive. The average fiber diameter and the fiber size distributions of each sample were analyzed by measuring the diameter of 100 random fibers from the captured SEM micrographs using an image analysis software (Image J, National Institutes of Health, Bethesda, MD, USA).

##### Fourier Transform Infrared Spectroscopy (ATR-FTIR)

ATR-FTIR spectra were performed in an ATR-FTIR spectrophotometer (Thermo-Nicolet is10 FT-IR Spectrophotometer, Waltham, MA, USA) to demonstrate the integration among the components used in the production of the dual-layer materials. Briefly, the samples were mounted directly on the sensor, and the spectra recorded over the range of 400–4000 cm^−1^ at a resolution of 4 cm^−1^.

##### Mechanical Strength Behavior

The tensile test was performed using a uniaxial tensile machine (DY-35 Adamel Lhomargy, Roissy en Brie, France), according to ASTM standard D3039/D3039M. Briefly, the dried rectangular specimens (40 mm × 10 mm (length × width)) were placed between two clamps separated by a distance of 1 cm, and a 100 N load cell was applied to the samples at a stretching rate of 2 mm/min until the break. On each sample, measurements were made five times at room temperature. The Young’s modulus (MPa) was determined by the slope of the initial linear portion of the stress–strain curve, and the tensile strength (MPa) and elongation at break (%) calculated according to the following equations (Equations (1) and (2)):

Tensile strength (MPa) = (Breaking force or the maximum load (N)/Cross-sectional area of the sample (mm^2^)) (1)

Elongation at break (%) = (The extension at the breaking point (mm)/Initial length of the sample (mm)) × 100(2)

All values were compared subsequently with the reported values of native human skin.

##### Wetting Studies

The surface wettability of the dual-layer materials was evaluated by the sessile drop method using a Dataphysics Contact Angle System OCAH-200. Briefly, distilled water droplets of a volume of 4 µL were dropped carefully onto the surface of each sample at different locations. The droplet landing was recorded by a high-resolution video camera attached to the analyzer within 20 s, and the contact angle measured by analyzing the shape of the drop. The average of the values was reported as mean ± S.D.

##### Porosity Assessment

The porosity of each layer of the produced dual-layer materials was measured using the liquid displacement method, as reported by Yeh et al. [40]. Briefly, the dry weight of the samples was recorded, as (W_s_). Then, the samples were immersed in a graduated cylinder with known volume (W_1_) of absolute EtOH for 40 min at 30 °C, and the amount of the ethanol after impregnation was refilled and weighted as W_2_. After removing the nanofibrous mats from the displacement liquid, the volume of the ethanol remaining in the graduated cylinder was recorded as W_3_. The dressing’s porosity was calculated as (Equation (3)):

(3) $$\mathrm{Porosity}\;\left(\%\right)=\frac{\left(W_{2}-W_{3}-W_{s}\right)}{\left(W_{1}-W_{3}\right)}\times 100$$

##### Water Vapor Transmission Rates (WVTR)

Water vapor transmission rates (WVTRs) were determined according to ASTM E96 (American Society for Testing Materials)—standard test method for water vapor transmission of the materials.

Briefly, circular samples were affixed to individual test tubes (diameter of opening = 1.2 cm) filled with 10 mL of distilled water and tightly sealed using parafilm tape along their periphery to prevent water loss. After that, the whole apparatus was weighed (W_i_) and incubated at 37 °C. After 24 h, the sample-glass tubes assembly was removed and weighed (W_f_), and WVTR calculated as outlined below (Equation (4)):

(4) $$\mathrm{Water}\;\mathrm{vapor}\;\mathrm{transmission}\;\mathrm{rate}\;\left(\mathrm{WVTR}\right)\;\left(g/m^{2}/\mathrm{day}\right)=\frac{W_{i}-W_{f}}{A}$$

where A is the exposure area (m^2^), and W_i_ and W_f_ are the initial and final weights of the test tubes, respectively.

##### Swelling and In Vitro Degradation Studies

The dried dual-layer materials were cut into 1 × 1 cm^2^ pieces, and pre-weighed (W_0_). These samples were soaked in 10 mL of PBS (pH = 5.5 and pH = 8.0) solutions at 37 °C and removed from the media at predetermined time points. Excess water on the surface was wiped with filter paper, and the swollen samples weighed (W_t_) immediately. The degree of water uptake was calculated as follows (Equation (5)).

(5) $$\mathrm{Water}\;\mathrm{Uptake}\;\left(\%\right)=\frac{W_{t}-W_{0}}{W_{0}}\times 100$$

Hydrolytic degradation was also monitored based on the weight change of the samples by immersing in PBS (pH = 5.5 and pH = 8.0) solutions at 37 °C. The percentage weight loss was determined based on the weight of the dried pre-weighed samples (W_0_) and the weight of dried samples after removing from the PBS solutions (W_d_), as follows (Equation (6)).

(6) $$\mathrm{Weight}\;\mathrm{loss}\;\left(\%\right)=\frac{W_{0}-W_{d}}{W_{0}}\times 100$$

#### 3.2.5. Study of the In Vitro AG Release

The AG release from the dual-layer materials was determined using 10 mL of PBS release medium at pH = 5.5 or pH = 8.0, with a shaking rate of 100 rpm in a thermostatic shaking incubator. At specific time points, samples were removed from the release medium, and an equal amount of fresh PBS medium was added. The concentration of AG was determined using a UV-visible spectrophotometer at a 400 nm wavelength, and the cumulative release of the AG calculated from a standard curve plotted with a known amount of AG as standard [22,41].

#### 3.2.6. Antibacterial Properties of the Dual-Layer Dressing Materials

The antibacterial activity of the produced dual-layer materials was tested according to the Standard Test Method for Determining the Activity of Incorporated Antimicrobial Agent(s) in Polymeric or Hydrophobic Materials (ASTM E2180-07 standard). Strains of *S. aureus* and *P. aeruginosa* were used. Briefly, agar slurry solutions (0.30% agar and 0.85% NaCl) were prepared and inoculated with overnight bacterial suspensions of ~10^8^ CFU/mL. Then, a thin semisolid layer of inoculated agar slurries was spread onto the surface of each sample, including a filter paper control (pore size of 0.22 µm), and allowed to gel. The test samples were incubated at 37 °C for 18–24 h for two different contact times (t = 0 h and t = 24 h). After each contact time, a sterilized saline solution (NaCl) was added to the falcon tubes containing the inoculated samples, and these were shaken vigorously to enable the release of the agar slurry. Next, serial dilutions were performed with NaCl solution, pipetted out, and spread onto agar plates. The plates were incubated at 37 °C for 18–24 h, and the log (CFU/mL) calculated for each sample.

#### 3.2.7. In Vitro Cytotoxicity Assay (MTT)

The cell viability and proliferation of the dual-layer materials were analyzed using a MTT assay as recommended by the ISO 10993-5:2009 guidelines (Biological evaluation of medical devices—Part 5: Tests for in vitro cytotoxicity). This involves direct cell contact with the material. Briefly, the NHDF cells were cultured in complete medium supplemented with fetal bovine serum (FBS) and incubated in a humidified incubator at 37 °C with 5% CO_2_. After cell seeding, the samples were cut into round disks to cover less than 10% of the well area, and then placed into a 24-well culture plate and sterilized by UV irradiation (254 nm, ≈ 7 mW cm^−2^) for 1 h. The NHDF cells were seeded onto the samples at a density of 1 × 10^4^ cells/well for 1, 3, and 7 days, and the medium changed every two days. At those specific time points, the medium was taken out, and the MTT reagent solution added to each well. The plate was covered with aluminized paper. After 4 h under the same conditions, the MTT solution was removed and the produced formazan dissolved in DMSO. The absorbance of each plate was determined by a spectrophotometric plate reader (BioRad xMark microplate spectrophotometer) with a test wavelength of 570 nm. Cells incubated without samples were used as a negative control (K^−^), and cells incubated with EtOH (96%) selected as a positive control (K^+^).

#### 3.2.8. Statistical Analysis

Statistical data analysis utilized one-way analysis of variance (ANOVA) followed by Tukey’s multiple comparison test using GraphPad Prism 6 software (GraphPad Software, La Jolla, CA, USA). Using a confidence level of ≥ 95%, values of *p*< 0.05 were considered statistically significant.

## 4. Conclusions

Chronic wounds and difficult-to-heal wounds continue to attract considerable interest due to associated high prevalence of bacterial infections. Based on aspirations to mitigate those risks, previous research has demonstrated the potential of wound dressing materials with antimicrobial activity. This study focused on contributing new insight regarding amalgamation of well-understood wound dressing physical properties with novel use of electrospun membrane containing an antimicrobial plant extract. The findings presented demonstrate potential to enhance skin regeneration while suppressing bacterial growth, complementing published data regarding analogous use of antibiotics, nanoparticles, and other natural compounds applied to electrospun nanofibers.

In summary, our novel dual-layer TEMPO-oxidized cotton/PVA_AG_CS dressing material exhibited attributes supporting its use for mechanical protection of wounds and physical barrier protection against infectious agents (i.e., albeit so far limited to *S. aureus and P. aeruginosa*). In addition, the coating layer of PVA_CS containing crude AG extract presented suitable morphology and achieved diameters within the size range of the collagen fibers in the natural skin (50–400 nm). Moreover, the TEMPO-oxidized cotton/PVA_AG_CS exhibited an adequate wettability, porosity, and swelling ration for a wound dressing. These characteristics are likely to enhance the therapeutic effectiveness of the produced material. Additionally, an initial burst release of AG in the first 6 h, followed by a sustained release profile, was also observed. Such data emphasize the suitability of the dual-layer TEMPO-oxidized cotton/PVA_AG_CS dressing material to maintain an aseptic environment at the wound site, which is crucial to avoid the wound bacterial colonization and infection.

## Figures and Tables

**Table 1:** Characterization of the mechanical properties of the produced dual-layer dressing material and associated raw materials.

	Tensile Strength (MPa)	Young’s Modulus (GPa)	Elongation at Break (%)	Thickness (mm)
**TEMPO-oxidized cotton**	36.98 ± 4.13	1.82 ± 0.25	2.04 ± 0.12	0.250 ± 0.011
**PVA_CS**	9.83 ± 16.38	0.41 ± 0.25	4.64 ± 1.40	0.051 ± 0.004
**PVA_AG_CS**	9.66 ± 1.95	0.35 ± 0.09	3.03 ± 0.60	0.012 ± 0.001
**TEMPO-oxidized cotton/PVA_CS**	22.48 ± 4.46	0.40 ± 0.14	5.80 ± 0.89	0.315 ± 0.011
**TEMPO-oxidized cotton/PVA_AG_CS**	26.55 ± 1.41	0.46 ± 0.11	5.95 ± 1.71	0.270 ± 0.004

**Table 2:** Classification of the toxicity level of the produced dual-layer dressing materials depending on the percentage of cell viability (%).

Standard Viability Data ^a^		Experimental Data			
Cell Viability (%)	Toxicity Level	Samples		Cell Viability (%)	Toxicity Level
≥100	0	**TEMPO-oxidized cotton**	Day 1	88.99	1
75–99	1		Day 3	98.50	1
50–74	2		Day 7	92.98	1
25–49	3	**TEMPO-oxidized cotton/PVA_CS**	Day 1	91.83	1
1–24	4		Day 3	105.79	0
0	5		Day 7	89.69	1
		**TEMPO-oxidized cotton/PVA_AG_CS**	Day 1	92.78	1
			Day 3	100.45	0
			Day 7	104.64	0

^a^ The standard viability data were obtained from reference [39].

## Data Availability

The data presented in this study are available in this article.
